# Cynanche Laryngea, or Acute Laryngitis

**Published:** 1838-02-14

**Authors:** N. Chapman

**Affiliations:** Professor of the Theory and Practice of Physic, in the University of Pennsylvania


### MEDICAL EXAMINER.

DEVOTED TO MEDICINE, SURGERY AND THE COLLATERAL SCIENCES.

**EDITED by J. B. BIDDLE, M. D. and M. CLYMER, M. D.**

No. 4. PHILADELPHIA, WEDNESDAY, FEBRUARY 14, 1838. Vol. I.

### CYNANCHE LARYNGEA,

### OR ACUTE LARYNGITIS.

*By* N. Chapman, M. D., *Professor of the Theory and Practice of Physic, in the University of Pennsylvania.*

(*Concluded from page* 43.)

It may be summarily stated, that Laryngitis is one of the most unmanageable diseases, sometimes resisting entirely our best efforts. Called early in the case, and pursuing a vigorous practice, it is cured, though with extreme difficulty, and still seldomer, or perhaps never, in the advanced stages, by medicine, where from neglect or inappropriate treatment, derangements about the glottis, or in the lungs, have been permitted to take place. The symptoms denoting favourably or otherwise, are sufficiently obvious without any further detail, from the history which I have given of the disease.

By the character of the case the appearances on dissection are varied. It has sometimes happened where death was sudden, probably from spasm only, no lesions were observable. But under other and more common circumstances, the lining of the larynx is met with turgid and red, the phlogosis diffused, or punctated, with the intervening spaces little or not at all changed. The sides of the rima glottidis often so approach, that the aperture is greatly diminished, or nearly closed, and the epiglottis, as I have said, is sometimes swollen and erected, leaving the glottis open or uncovered. Extravasations on the surface of the membrane seldom take place, though instances are mentioned of a collection of mucus—still more rarely of pus, and occasionally of a slight pellicle of lymph spread over it. The proper adventitious membrane of Croup never exists.

Cutting through the mucous membrane, a copious serous, or lymphy, or purulent effusion into the subcellular tissue is sometimes met with. Nor are these fluids uniformly confined to this immediate position. The inflammation may be more pervading, and so intense, that even abscesses are formed in remoter parts of the cellular tissue, of which Armstrong gives an example, of one between the muscles of the pharynx, and the bodies of the cervical vertebræ. These are the appearances in, or about, the larynx.

The trachea is seldom involved, and certainly not to any extent. No examples indeed, says a late writer, are on record of the inflammation having descended beyond the larynx. This is not however, exactly the fact. Leveille relates a case where it was manifested throughout the windpipe to its extremest ramifications.* The Bronchiæ are occasionally choaked up by an excess of glairy or more viscid secretions, and the cellular tissue of the lungs edematous, or they are loaded with dark blood, or inflamed in their substance or pleural coverings. It is hardly necessary to advert to the appearances in the throat when the case proceeds from an extension of the phlogosis of that part, they being such as belong to common or other states of tonsilitis, and its complications.

Embarrassment as to the pathology of laryngitis is still confessed. Enough, however, is ascertained, to assure us of its seat being mainly in the upper portion of the windpipe; and that the action partakes of a mixture of spasm, and inflammation, is no less apparent. But, in this respect, there is a striking similitude to Croup in the early stage, then, the most remedial of diseases,—whereas Laryngitis proves directly the reverse! To solve this problem satisfactorily, has perplexed some of the ablest writers. Towards an explanation of it, I will merely suggest, that much of the peculiarity of the latter affection, as well in relation to the symptoms, as the difficulty of the cure proceeds from the position and nature of the phlogosis.

That of Croup, restricted more especially to the mucous tissue, is adhesive, eventuating usually in the exudation of coagulable lymph on its surface. But in Laryngitis, expending its force chiefly on the sub-cellular membrane, it is of another character, productive of tumefaction, ending sometimes very speedily, and often when protracted, in effusion of serum, or lymph, or the secretion of pus, the first being the most common event. Laryngitis, in a word, is an affection, having an identity with the inflammation of common sore throat, in every leading feature, and is more serious only, from its location in a more vital structure. It is very intelligible how either of its ultimate states I have mentioned, influences the case. By swelling from phlogosis alone, the Larynx may be obstructed, and to this condition, the peculiar phenomena of the affection are mostly referrible. But it is occasionally otherwise. The cellular which lies under the mucous membrane, becoming distended by an

*Gazette de Santé, 1827.

effusion, pressure is made on the rima glottidis, approximating its sides, thus occasioning a similar mechanical interception to the passage of air to the lungs.

Considering the difference of condition in the two diseases, in the early stage, ought we to be surprised at the greater intractability of Laryngitis? An inflammation of the cellular membrane, it is well known, is never easy of resolution, proceeding most frequently to some other termination, in spite of our endeavours to arrest it; and in relation to the intumescence from extravasated fluids, we must be equally aware, that the customary remedies in Croup cannot succeed. This is one of the many instances in which the doctrine of tissues, beautifully illustrates the obscurest points of pathology, guiding us at the same time to a more correct and enlightened practice.

There is only another consideration to which I deem it necessary to allude. We have seen that Croup and this disease, though occupying nearly the same position, occur mostly at different periods of life, and that the adventitious membrane is peculiar to the former affection. An explication of this difference may perhaps be attained on physiological principles. During the growth of the body much fibrin is required in the formation and increase of organs, and accordingly, it abounds in the blood. But on the completion of this process, it diminishes, and in advanced age, bears a less proportion to the serum, which now exists in similar excess.

To overcome the phlogosis I have described, so as to prevent suffocation from the closing of the glottis by it or the edematous state subsequently induced, is the great object in the treatment of Laryngitis.

The chief remedy in the beginning, consists in copious venesection, urged sometimes, even ad deliquium animi. Less extensively used, it is altogether inadequate to an extreme emergency. What was formerly remarked of venesection, in *abating* or subverting action, applies *a fortiori* for reasons there assigned, to the case before us. Entirely persuaded am I, that much of the want of success complained of in this disease, is to be ascribed to the insufficient bleedings usually practiced in it. Most of the European physicians commend the practice, and pursue it, though it appears from the history of the cases reported, with few exceptions, not with intrepidity, and scarcely ever to the extent I have suggested. Conceding the decided efficacy of such a large loss of blood in Croup, about which there can be no longer any controversy, it surely is equally or more demanded in Laryngitis, and what might thus be presumed, has been verified on trial. The only cases of the disease I have ever cured, or seen cured, were mainly by this energetic practice. Nor in reference to the ends in view, is scarcely any delay admissible in the application of it. Even in a few hours, effusion may take place, and then it will be nugatory, and, perhaps detrimental, by exhausting the vital forces, without making any impression on the lesion itself. The language of Macbeth, on another occasion, conveys to us here a useful lesson:

"If it were done, when 'tis done, then 'twere well
It were done *quickly*."

It was probably on this account, the smallness of the bleeding, that Baillie was led to question its efficacy, in which estimate, several of the very respectable foreign writers seem to coincide. Washington's death, humanly speaking, may be ascribed to his having been so sparingly bled in the very commencement of the attack. The subsequent and larger bleedings were too late, effusion having taken place. He was a very robust man, of a sanguineous temperament, in whom such an inflammatory attack required the freest depletion.

Laryngitis, it is alleged, often occurs in aged persons, and sometimes, with shattered constitutions, who cannot bear this practice. But, what is there, that can be substituted with any hope of success? Even if some risk may be incurred, which I do not think can be, under any circumstances, from a single bleeding, however large, it is surely better to venture it, than to permit the patient to perish unaided, from any dread of responsibility. An enormous loss of blood, however, is not always exacted, the disease ordinarily yielding to a more moderate quantity.

As soon as the operation is over, we are to endeavour to excite vomiting, by a combination of tartarized antimony, ipecacuanha, and calomel, the action of which, may be promoted by the warm bath. The late Dr. Armstrong has given some very strong evidence to the utility of the emetic practice, which, indeed, he seems to have preferred to every measure. Disappointed in the results of blood-letting general and local, he was induced to try active vomiting in several cases, which subsequently came under his care. "No circumstance in my life," says he, "ever gratified me more, than the great and sudden relief which vomiting afforded; in reality, it removed all the urgent symptoms at the time, and being repeated as soon as the slightest signs of stricture in the Larynx returned, at last completed the recovery."

As well from actual experience in this application of them, as the analogy of their decided efficacy in Croup, and still more in the phlegmasia of the throat, which I have said bears the closest affinity to Laryngitis, I am disposed to appreciate highly emetics, in this case. But though undoubtedly beneficial, there is some extravagance in the commendation of them I have quoted, and, to direct them to the exclusion of venesection, or in any other view than in subordination to it, I am sure must be unwarrantable.

The disease not submitting, we are next to resort to leeches to the throat, then to emollient poultices, and finally to a blister, with inhalations of the mildest vapours.

Evacuations by calomel, I have found useful.

As the disease is characterised by recurrences of violent paroxysms of spasm, the antispasmodics have been suggested, which possibly might be serviceable on the proper reduction of action, though the practice strikes me as equivocal, and hence I have never directed it.

Nothing within my experience, and I have tried diverse means, so completely controls these periodical exasperations as a cataplasm of tobacco around the neck, or smoking the article, where the individual is not habituated to its use. The success indeed, of the few experiments I have made with it, leads me to believe, that it may prove a very important therapeutic agent in the more general management of the disease. It occasions very severe and protracted nausea, followed by thorough relaxation, and probably abatement of inflammatory action.

The period having arrived, when the directly depleting measures can be carried no further, I resort to sweating, by the Dover's powder, freely given, and the vapour bath. This process ought to be continued for several hours unremittingly, and thus conducted, I may say, from the experience of several years, deserves much attention. Baffled sometimes as formerly stated, in my efforts to relieve the advanced stage of croup, I originally resorted to it in that affection, and encouraged by the occasional results, was led by analogy to extend the practice to this disease, where it has proved more uniformly effectual as I expected, from the difference of the pathological condition in the two cases, the existence usually of the false membrane in the one, constituting a peculiar and insuperable obstacle to success.

This is, perhaps, the best that can be effected in this stage of the disease, and when more advanced, our resources become exceedingly limited, and will be found I apprehend, so precarious, as to deserve for the most part, little confidence. It is proper, at such a conjuncture, to endeavour, as prelusive to any course of treatment, to determine the exact state of things,—whether it be owing to swelling from inflammation in the larynx, or to edema of its cellular tissue, or to congestion or other morbid states of the lungs. These are considerations which can alone guide us to correct and definite practice, and which may be determined by a comparison of symptoms, and still more precisely by the external means of exploration. But of the conditions predicated, the first, or that regarding the larynx itself, can now only claim to be noticed. The affections of the lungs have in part already been disposed of, and the rest will hereafter be fully treated.

Entertaining an impression of a still enduring inflammation, either in the mucous or sub-cellular tissue of the larynx, a repetition of local bleeding, to be succeeded by further counter-irritations, promises most. Expectorants, so serviceable in somewhat similar affections, are here of no advantage, except, perhaps, the antimonial preparations, so given as to operate merely on an antiphlogistic principle. My chief reliance would be placed on a combination of calomel, opium, and ipecacuanha, not so much from any actual knowledge of its utility, in this particular application of it, as its confessed efficacy in the cure of reduced states of inflammation generally.

An allusion has been made to a variety of the disease, where even in the commencement, in place of activity of inflammation, an edematous disposition is manifested in the throat extending down into the larynx. Cases of this sort, I have reason to believe, are peculiar to the lymphatic temperament, and should be managed very differently from ordinary laryngitis. Excepting by leeches, and these only, in the earliest stage, and moderately, the loss of blood is not admissible. Emetics here are particularly serviceable, a blister to the neck scarcely less so, and I have derived advantage from touching the whole of the fauces with a strong solution of lunar caustic, and still more, with the powder of burnt alum. These applications, especially the latter, produce a powerful impression, reaching even to the larynx, subversive of the preëxisting state of the parts, and thus arresting the further progress of the disease. They are, in every view, preferable to the stimulating gargles which have been recommended, and ordinarily employed.

Effusion having taken place, and life menaced, it seems to me, that by cutting down into the cellular membrane, or perhaps, by simple puncture, the fluid might be made to escape, and relief consequently afforded. But this suggestion, so far as I know, has never been carried into execution, at which I am not a little surprised, as I really do not perceive any objection to such an operation. It affords me, however, gratification to find, that it has lately received the sanction of Lisfranc, whose authority is indisputable.

As a dernier alternative in either case, laryngotomy has commonly been proposed. It was strongly advised by Baillie, the opinions of whom are always to be respected. To be useful however, it must be properly timed. Too early performed, it can hardly fail, while active inflammation prevails, to become an aggravating cause, and too long delayed, or till structural changes take place in the larynx itself, or the lungs are seriously implicated, or the sinking condition supervenes, it proves altogether nugatory.

It will be right, prior to attempting the operation, to endeavour to ascertain the state of the parts with a view to the determination of its propriety, and this so far as regards the lungs, may be done by the means just indicated.

By opening the wind-pipe in due season, respiration would proceed in spite of the obstruction of the glottis, the irritated structures restored to quiescence, or at least, relieved from the existing violent agitation, so exasperating in its effects, and which by continuance, must produce pulmonary implication, or effusion into the cellular tissue of the larynx itself. From the wound, the danger is in no respect enhanced. The aperture is to be allowed to remain open, until the inflammation subsides, and the natural passage re-established, by the subsidence of the tumefaction, or the removal of other impediments.

As practised ordinarily, the operation is very defective. The opening without a canula closes, and, therefore is abortive. To introduce, as well as to retain that instrument is difficult from the spasmodic movements it excites, and as an extraneous body, it must be very mischievous, in the highly irritable state of the parts. It does indeed appear, that in several instances, where there was the fairest chance of the operation succeeding, it proved otherwise, suffocation taking place from the immense accumulation of mucus, owing in all likelihood to the irritation thus induced.

Convinced of the force of these objections, the late Professor Physick early abandoned the use of the canula, and kept open the aperture by means of a very ingenious contrivance. Carmichael* an eminent surgeon of Dublin, also operates in a new mode, and with entire success, in the only case in

*Transactions Dublin, C. M. Vol. iv. 311 p.

which he has had an opportunity of trying it. To the lectures on Surgery I refer you for an account of these improved operations.

Laryngotomy has been three times performed in this city by Dr. Physick, once in Croup, and twice in Laryngitis. But on each occasion, in the very last stage of the disease, and consequently without success. No relief was afforded in two of the cases owing to lesions of the lungs, and death speedily ensued. But in the third Laryngitis, there was such a complete suspension of all the distressing symptoms for several hours, as to afford hope of recovery, though a sudden return of spasm terminated it otherwise. The operation has also been performed by Dr. Rhea Barton in a case of Croup, which I attended in consultation with Dr. Jackson, and here likewise, it proved unavailing. The child however was *in extremis*, and a tubular membrane was found reaching from the top of the larynx to the ramifications of the bronchiæ. Great portion of it, being detached and brought away by the forceps, an inexpressible degree of relief was afforded, for a considerable length of time, and the child would probably have done well, had it not been for an exorbitant secretion of mucus, by which it was ultimately stifled.

Notwithstanding these failures, I cannot forbear to press upon you the importance of this resource of our art. Contemplating the two diseases, we shall be led to the conclusion that it promises much more in this than in cynanche trachealis. The former is comparatively seldom extended to the lungs, is exempt from the lymphy extravasations, or membranous formations in the larynx, and hence the operation is incomparably less liable to miscarriage. What thus appears *a priori*, experience confirms. The operation has been resorted to, in probably about an equal number of instances of the two diseases, though with much better effect in Laryngitis. Twenty eight well authenticated cases of success have I collected, of which, nineteen were in the latter, and eleven in the former affection. But I repeat that it is not too long to be postponed. Convinced as I am by the most melancholy results, of the too frequent intractability of this affection, in its second stage, to any course of medical treatment, I would not hesitate, seeing its unrelenting progress, to recur at once to the operation as the only means of preserving life. Delayed till an emergency shall arise, which may seem to warrant, as a final resource, this proceeding, in the estimation of friends and attendants, we lose the golden season, and must be prepared to encounter the odium of an unfortunate attempt, with the uncomfortable reflection of what we had sacrificed by irresolution to prejudices, which ought never to be consulted, against the suggestions of judgment, or the dictates of duty.

